# Postoperative MRI Artifact Burden After Carbon Fiber-Reinforced PEEK Versus Titanium Instrumentation in Metastatic Spinal Disease

**DOI:** 10.3390/cancers18142199

**Published:** 2026-07-08

**Authors:** Kamil Krystkiewicz, Aleksander Kowal, Agata Krajniak, Michał Dziatosz, Mateusz Bilski, Łukasz Kuncman, Marcin Tosik

**Affiliations:** 1 Department of Neurosurgery and Neurooncology, Copernicus Memorial Hospital in Lodz, 93-513 Lodz, Poland; aleksanderwkowal@gmail.com (A.K.); agata.krajniak@gmail.com (A.K.); dziatosz.michal@hotmail.com (M.D.); marcin.tosik@wp.pl (M.T.); 2 Department of Radiotherapy, Affidea Nu-Med Center of Oncology Diagnostics and Therapy, 22-400 Zamosc, Poland; bilskimat@gmail.com; 3 Department of Radiotherapy, Medical University of Lublin, 20-093 Lublin, Poland; 4 Department of Radiotherapy, Medical University of Lodz, 93-513 Lodz, Poland; lukaszkuncman@gmail.com; 5 Department of External Beam Radiotherapy, Copernicus Memorial Hospital in Lodz, 93-513 Lodz, Poland

**Keywords:** spinal metastases, carbon-fiber implants, titanium instrumentation, postoperative MRI, MRI artifacts, oncologic spinal instrumentation MRI artifact score

## Abstract

Background/Objectives: Patients undergoing surgery for metastatic spinal disease often require instrumented stabilization followed by radiotherapy and postoperative magnetic resonance imaging (MRI) surveillance. Titanium implants may generate artifacts that impair assessment of the operated level, whereas carbon fiber-reinforced polyetheretherketone (CFR-PEEK) constructs may improve MRI assessability. This study compared CFR-PEEK and titanium instrumentation with respect to postoperative MRI artifact burden, early outcomes, and oncological follow-up, and introduced a study-specific artifact grading system. Methods: This retrospective single-center cohort included 78 patients treated with instrumented stabilization for metastatic spinal disease: 33 with CFR-PEEK and 45 with titanium instrumentation. Postoperative MRI suitable for artifact assessment was available in 47 patients. Implant-related artifacts were evaluated using the Oncologic Spinal Instrumentation MRI Artifact Score (O-SIMAS), a 0–5 anatomy-based grading system. Early postoperative outcomes, local recurrence, progression-free survival, overall survival, and interrater agreement were analyzed. Results: CFR-PEEK instrumentation was associated with lower postoperative MRI artifact burden than titanium instrumentation, with median O-SIMAS scores of 2.0 versus 3.0, respectively (*p* < 0.001). High-grade artifacts were less frequent after CFR-PEEK than after titanium instrumentation (15.8% versus 67.9%; *p* = 0.0008). Titanium instrumentation remained independently associated with high-grade artifacts. Early postoperative outcomes, local recurrence, progression-free survival, and overall survival did not differ significantly between groups. O-SIMAS showed substantial pairwise interrater agreement. Conclusions: CFR-PEEK instrumentation was associated with improved postoperative MRI assessability and fewer diagnostically relevant artifacts than titanium instrumentation. No oncological benefit was shown. O-SIMAS may support structured artifact assessment, but requires external validation.

## Simple Summary

Patients with cancer that has spread to the spine often need surgery with implants to stabilize the spine, followed by radiotherapy and magnetic resonance imaging surveillance during follow-up. Standard titanium implants can cause image distortions that make it harder to assess the operated area and detect possible tumor recurrence. Carbon fiber-reinforced implants may reduce these distortions and improve postoperative imaging. In this study, we compared carbon fiber-reinforced and titanium implants with respect to postoperative magnetic resonance imaging assessability, implant-related artifacts, and early clinical outcomes in patients treated surgically for metastatic spinal disease. We also introduced a structured scoring system to describe how much implant-related distortion affects the assessment of postoperative magnetic resonance images. Carbon fiber-reinforced implants were associated with fewer clinically relevant imaging artifacts than titanium implants, while early clinical outcomes were similar. These findings suggest that carbon fiber-reinforced implants may improve postoperative imaging assessment in spinal oncology, although larger studies are needed to validate the scoring system and confirm its clinical usefulness.

## 1. Introduction

Surgical treatment of metastatic spinal disease often requires instrumented stabilization to restore spinal stability, reduce pain, and facilitate neural decompression [1,2,3]. In contemporary spinal oncology, surgery is frequently integrated with postoperative radiotherapy and MRI-based surveillance, particularly in patients treated with separation surgery principles and stereotactic body radiotherapy strategies [4,5,6,7,8,9,10,11,12]. In this setting, implant material may influence not only the mechanical aspects of reconstruction but also the quality of postoperative imaging and the reliability of assessment of residual or recurrent disease at the operated site.

Titanium instrumentation remains widely used in spinal oncology because of its established mechanical performance and availability. However, titanium implants can produce substantial MRI artifacts and may also interfere with postoperative radiotherapy planning by reducing the accuracy of target and organ-at-risk delineation [13,14,15,16,17,18]. Carbon fiber-reinforced polyether ether ketone (CFR-PEEK) instrumentation has been introduced as a radiolucent alternative with reduced artifact susceptibility. Previous clinical and experimental studies have suggested that CFR-PEEK constructs may improve postoperative MRI assessability and facilitate radiotherapy planning while maintaining acceptable early clinical performance [19,20,21,22,23,24,25,26,27,28].

Despite these advantages, the available evidence remains limited. Many studies describe implant-related MRI artifacts qualitatively or classify postoperative imaging as assessable or non-assessable, without a structured description of which anatomical regions are affected. This distinction is clinically relevant in metastatic spinal disease because artifacts may selectively impair evaluation of the epidural space, spinal canal, operated vertebral body, or reconstruction site, all of which are important for postoperative surveillance and detection of residual or recurrent tumor [13,14,15,16,17,18,28]. Moreover, there is no widely adopted grading framework for describing the anatomical extent and diagnostic relevance of postoperative implant-related MRI artifacts in spinal oncology. Existing frameworks for screw placement accuracy and response assessment after spine stereotactic body radiotherapy address related but distinct problems and do not specifically grade implant-related MRI artifact burden [29,30].

To better contextualize the present study, key previous studies evaluating CFR-PEEK instrumentation in spinal oncology are summarized in Table 1. These studies consistently suggest potential advantages of CFR-PEEK for postoperative imaging and radiotherapy planning, while also highlighting the limited availability of comparative clinical data and the lack of a structured, anatomy-based MRI artifact-grading framework specific to spinal oncology.

Overall, previous studies have consistently suggested that CFR-PEEK instrumentation may improve postoperative imaging assessability and facilitate radiotherapy planning in spinal oncology. However, most available studies have focused on feasibility, safety, clinical outcomes, or broad qualitative descriptions of imaging advantages. Comparative data specifically addressing postoperative MRI artifact burden remain limited, and artifact assessment has often been reported using qualitative or binary categories rather than a structured anatomical grading framework. In particular, no widely adopted scale has been established to describe whether implant-related artifacts compromise the epidural space, spinal canal, operated vertebral body, or reconstruction site, which are the key regions assessed during postoperative surveillance for residual or recurrent disease.

Taken together, the available literature identifies an unresolved need for a comparative, anatomy-based assessment of postoperative MRI artifacts after CFR-PEEK versus titanium instrumentation in patients with metastatic spinal disease. Accordingly, the present study was designed to address this gap by comparing CFR-PEEK and titanium instrumentation with respect to postoperative MRI artifact burden and assessability at the surgical level in patients treated surgically for metastatic spinal disease. We also introduced the Oncologic Spinal Instrumentation MRI Artifact Score (O-SIMAS), a preliminary, study-specific, anatomy-based grading system intended to describe the anatomical extent and diagnostic relevance of implant-related MRI artifacts. Secondary and exploratory aims included comparisons of early postoperative outcomes, local recurrence, progression-free survival, and overall survival. We hypothesized that CFR-PEEK instrumentation would be associated with lower postoperative MRI artifact burden and fewer high-grade artifacts than titanium instrumentation, while early clinical outcomes would be comparable between groups.

## 2. Materials and Methods

### 2.1. Study Design and Patient Population

We conducted a retrospective, single-center cohort study of consecutive patients who underwent surgery for metastatic spinal disease at the Department of Neurosurgery and Neurooncology at Copernicus Memorial Hospital in Lodz, Poland, from 2022 to 2025. The study was reported in accordance with the Strengthening the Reporting of Observational Studies in Epidemiology (STROBE) statement for cohort studies (Supplementary Material Table S1).

During the study period, 109 consecutive patients underwent surgery for metastatic spinal disease at our institution. Of these, 79 patients received instrumented stabilization. Patients treated without instrumentation, those with missing implant material data, and one patient treated with a mixed construct containing both carbon fiber-reinforced polyetheretherketone (CFR-PEEK) and titanium components were excluded from the comparative analysis. The final comparative cohort therefore included 78 patients treated with either purely CFR-PEEK or purely titanium instrumentation.

This cohort of 78 patients was used to analyze baseline characteristics, early postoperative outcomes, local recurrence, progression-free survival, and overall survival. Postoperative magnetic resonance imaging (MRI) suitable for assessing implant-related artifacts was available in 47 of these 78 patients. Among the 47 MRI-evaluable patients included in the primary artifact-burden analysis, 33 originated from the randomized CarboMetaSpine trial, whereas 14 were treated outside the trial pathway. Therefore, although most patients in the MRI-evaluable subgroup were drawn from the randomized trial cohort, the artifact analysis should still be interpreted as a retrospective secondary analysis, as the overall study population also included non-randomized patients. This MRI-evaluable subgroup was used for the primary artifact-burden analysis with the O-SIMAS. Among the 31 patients without postoperative MRI available for artifact assessment, 15 died before a planned or clinically indicated postoperative MRI could be performed. In the remaining cases, postoperative MRI was unavailable because of loss to follow-up or inability to contact the patient after discharge. Thus, the absence of postoperative MRI was mainly related to early mortality and incomplete follow-up rather than to predefined imaging-based exclusion criteria. Because MRI availability was not uniform across the cohort, artifact-related analyses were restricted to patients with available postoperative MRI and should be interpreted in the context of potential selection bias.

Implant allocation was not uniform across the cohort. Since 2023, our institution has been recruiting patients for the CarboMetaSpine trial (ClinicalTrials.gov identifier: NCT06293157) [31], in which eligible patients with metastatic spinal disease planned for surgical stabilization and stereotactic body radiotherapy (SBRT) were randomized to receive either CFR-PEEK or titanium instrumentation according to the trial protocol. In the present cohort, 35 patients were randomized within the CarboMetaSpine trial. Of these 35 randomized patients, 33 had postoperative MRI suitable for artifact assessment and were therefore included in the MRI-evaluable subgroup. The remaining patients were not randomized because they did not meet the trial inclusion criteria, were treated before trial enrollment was available, or were managed outside the trial pathway. In these non-randomized cases, the treating multidisciplinary team chose between CFR-PEEK and titanium instrumentation based on implant availability, spinal anatomy, mechanical stability requirements, extent of reconstruction, anticipated need for postoperative MRI surveillance, and radiotherapy planning considerations. Therefore, although a substantial proportion of the cohort was randomized, implant allocation in the overall retrospective cohort remained partially non-randomized, and residual selection bias cannot be excluded. The randomized CarboMetaSpine trial component was conducted in accordance with the Declaration of Helsinki and was approved by the Bioethics Committee at the Polish Mother Health Center Institute in Lodz (approval no. 90/2022 from 20 September 2022, with extensions no. 69/2024 from 18 June 2024 and no. 53/2025 from 17 June 2025). Patients enrolled in the CarboMetaSpine trial provided written informed consent in accordance with the trial protocol. For the retrospective analysis, only previously collected and anonymized clinical data were used, and no additional diagnostic, therapeutic, or experimental intervention was performed.

### 2.2. Data Collection

Clinical, radiographic, oncologic, and perioperative data were retrospectively collected from the institutional database and source medical records. Extracted variables included age, sex, primary tumor diagnosis, neurological status, admission mode, surgical technique, implant material, postoperative radiotherapy, postoperative MRI findings, local recurrence, postoperative complications, reoperation, follow-up duration, and survival status.

Postoperative radiotherapy for the operated spinal level was categorized as no postoperative radiotherapy, conventional or other non-stereotactic radiotherapy, or SBRT. Local recurrence was defined as radiologically documented tumor recurrence or progression at the level operated on during follow-up.

### 2.3. MRI Artifact Assessment

Postoperative implant-related artifacts were assessed using the O-SIMAS, an original, author-developed, study-specific grading system. O-SIMAS was created for the present study and has not been previously published or externally validated. The score was designed by mapping implant-related artifacts to the anatomical regions most relevant to postoperative assessment in spinal oncology. These regions included bone adjacent to the pedicle screws, the epidural space adjacent to the instrumentation, the epidural space at the operated level, the spinal canal, the operated vertebral body, and the vertebral body reconstruction site. Higher grades were assigned when artifacts extended from screw-adjacent regions to structures that are more directly relevant for assessment of residual or recurrent tumor. Thus, the score was intended to describe not only the presence of artifact, but also whether its anatomical location limited clinically meaningful postoperative MRI interpretation.

**Figure 1:**
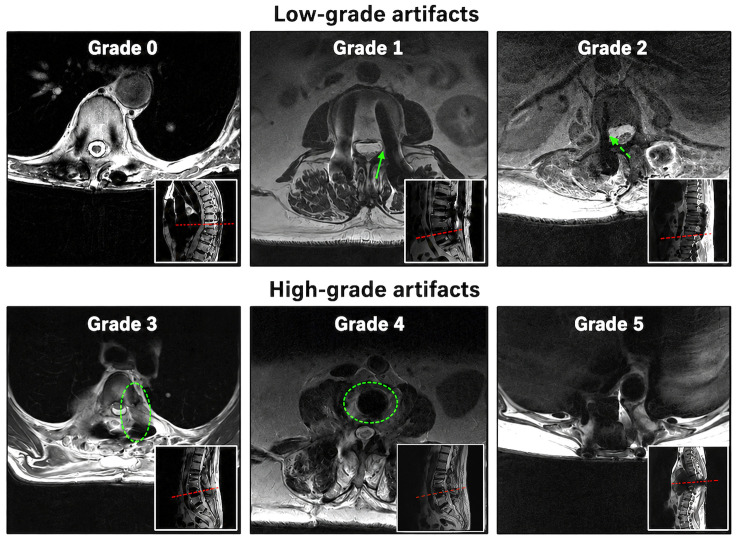
Anatomy-based radiologic grading scale for postoperative MRI artifact burden. Representative MRI examples illustrating the six-grade artifact scale. Each panel shows an axial MRI image with a sagittal inset. The upper row presents low-grade artifacts: Grade 0, no artifact limiting diagnostic assessment; Grade 1, green arrow—artifact around pedicle screws limiting assessment only of bone adjacent to the screws; and Grade 2, green arrow—artifact limiting assessment of the epidural space adjacent to the instrumentation, without extending beyond the mid-disc line between the instrumented vertebra and the operated vertebra. The lower row presents high-grade artifacts: Grade 3, artifact limiting assessment of the epidural space at the operated level, defined as artifact extending to or below the mid-disc line; Grade 4, artifact limiting assessment of the operated vertebral body or vertebral body reconstruction site; and Grade 5, artifact limiting assessment of both the epidural space at the operated level and the operated vertebral body or vertebral body reconstruction site. Green dashed lines and objects indicate the artifacts, and red dashed lines indicate the axial cut of the spine.

The O-SIMAS grades, illustrated by representative examples in Figure 1, were defined as follows: grade 0, no diagnostically relevant artifact; grade 1, artifact limited to bone adjacent to pedicle screws; grade 2, artifact affecting the epidural space adjacent to the instrumentation but not extending beyond the mid-disc line between the instrumented vertebra and the operated vertebra; grade 3, artifact affecting the epidural space at the operated level, defined as extension to or below this mid-disc line; grade 4, artifact limiting assessment of the operated vertebral body or vertebral body reconstruction site; and grade 5, artifact limiting assessment of both the epidural space at the operated level and the operated vertebral body or reconstruction site. Figure 1 visually demonstrates the anatomical rationale of the O-SIMAS grading system, showing the transition from low-grade artifacts limited to screw-adjacent bone or adjacent epidural space to high-grade artifacts that compromise assessment of the epidural space at the operated level and the operated vertebral body or reconstruction site.

O-SIMAS was not intended to represent a biologically linear severity scale. Rather, it was designed as a pragmatic, anatomy-based framework in which artifact grades are assigned based on the anatomical structures obscured by implant-related distortion. The rationale was that artifacts confined to screw-adjacent bone are less likely to compromise postoperative oncological assessment, whereas artifacts involving the epidural space at the operated level, the spinal canal, the operated vertebral body, or the reconstruction site may directly limit evaluation of residual or recurrent tumor. Therefore, O-SIMAS prioritizes the location and diagnostic consequence of the artifact over artifact size alone. In practical terms, a relatively small artifact may receive a higher grade if it obscures a clinically critical region, such as the epidural space at the operated level or the vertebral body reconstruction site, whereas a larger artifact may receive a lower grade if it remains confined to screw-adjacent bone and does not impair assessment of the operated level. The distinction between grades 3 and 4 is anatomical rather than hierarchical, with grade 3 primarily involving the epidural compartment at the operated level and grade 4 involving the operated vertebral body or reconstruction site. Accordingly, the full 0–5 score should be interpreted cautiously until external validation is available.

Postoperative MRI examinations were performed on a 1.5-T MRI system (Vantage Orian; Canon Medical Systems Corporation, Otawara, Tochigi, Japan) using a standardized institutional postoperative spine MRI protocol. The protocol included sagittal and axial T1-weighted, contrast-enhanced T1-weighted, T2-weighted, short tau inversion recovery, and fat-suppressed sequences, as well as diffusion-weighted imaging and corresponding apparent diffusion coefficient maps. Axial images were planned at the operated level and across the instrumented segment, whereas sagittal images covered the operated and adjacent spinal levels. In the standard protocol, sagittal sequences were typically acquired with a slice thickness of 4.0 mm and a slice spacing of 4.8 mm; for the sagittal T2-weighted sequence, representative acquisition parameters included a field of view of 250 × 300 mm, a matrix of 384 × 288, and TR/TE of 4458/120 ms. Metal artifact reduction techniques available on the scanner, including metal artifact reduction sequences and optimization of acquisition parameters, were used when assessing the instrumented spinal segment. Minor protocol adjustments were permitted based on the anatomical region, the extent of instrumentation, and patient-specific technical requirements.

In the MRI-evaluable subgroup, implant-related MRI artifacts were assessed on axial and sagittal images at the operated level using the O-SIMAS scale. For each patient, raters assigned a grade from 0 to 5 according to the most diagnostically limiting artifact observed at the operated level. The assessment focused on the anatomical extent of the artifact and its relevance for evaluating the epidural space, spinal canal, operated vertebral body, and reconstruction site. In patients with more than one postoperative MRI examination, the first MRI suitable for artifact assessment was used for the primary analysis.

Postoperative MRI examinations were independently reviewed by three raters with 10, 5, and 5 years of experience in oncologic spine imaging, respectively. The raters were blinded to clinical outcomes. Complete blinding to implant material was not feasible because implant type could often be inferred from imaging appearance and artifact pattern. Interrater agreement was calculated using the original independent ratings before any consensus discussion. For the primary artifact analysis, a final consensus O-SIMAS grade was assigned after review of discrepant cases. Cases with disagreement exceeding one grade or disagreement crossing the prespecified low-grade/high-grade threshold were resolved by consensus. The consensus O-SIMAS grade was used for group comparisons and regression analyses.

### 2.4. Dichotomized Artifact Classification

For secondary analyses, O-SIMAS was dichotomized into low-grade and high-grade artifact categories. Grades 0–2 were classified as low-grade because the artifact did not compromise the key anatomical structures at the operative level most relevant to postoperative tumor assessment. Grades 3–5 were classified as high-grade because the artifact impaired assessment of clinically critical structures at the level of operation, including the epidural space, spinal canal, operated vertebral body, or reconstruction site.

The threshold between grades 2 and 3 was selected on anatomical and clinical grounds. Grade 2 represents artifacts that affect the epidural space adjacent to the instrumentation but remain above the mid-disc line between the instrumented and operated vertebrae. In this situation, the artifact is close to the hardware but does not reach the operated level itself. In contrast, grade 3 represents artifact extension to or below this mid-disc line, meaning that the artifact reaches the epidural space at the operated level. This boundary was chosen because the epidural space at the level of operation is a key region for postoperative assessment of residual or recurrent tumor. Therefore, artifacts crossing this threshold were considered more likely to compromise clinically meaningful MRI interpretation and were classified as high-grade. Because O-SIMAS is an author-developed scale, this dichotomization should be regarded as study-specific and hypothesis-generating rather than formally validated.

In cases involving polymethylmethacrylate-augmented pedicle screws, the cement was considered part of the implant construct and assessed as an extension of the screw. This approach was considered clinically and radiologically justified and consistent with the anatomical logic of the grading system.

### 2.5. Outcomes

The primary endpoint was postoperative MRI artifact burden at the operated level, assessed using the study-specific O-SIMAS scale in the MRI-evaluable subgroup. Secondary endpoints included dichotomized artifact severity (low-grade versus high-grade artifact); limitation of postoperative MRI assessment due to implant-related artifact; local recurrence; new postoperative neurological deficit; wound-healing complication; reoperation; and length of hospital stay.

Exploratory time-to-event endpoints included progression-free survival and overall survival, both calculated from the date of surgery. Progression-free survival was defined as the interval from surgery to local recurrence or death, whichever occurred first. Local recurrence was additionally analyzed in a competing-risks framework, with death without prior local recurrence treated as a competing event.

### 2.6. Follow-Up

Follow-up data were obtained from postoperative clinical and imaging records. For patients with documented local recurrence, the event date was defined as the date of recurrence documented in the radiological record. Patients without local recurrence were censored on the date of the last documented follow-up or the last available clinical or radiographic assessment. For overall survival analyses, patients who were alive at the last follow-up were censored at the date of last contact.

### 2.7. Statistical Analysis

Continuous variables were presented as mean ± standard deviation or median with interquartile range, as appropriate. Categorical variables were presented as counts and percentages. For comparisons between two independent groups, continuous variables were analyzed using the Student *t*-test when normally distributed, or the Mann–Whitney U-test when not normally distributed or ordinal. Categorical variables were compared using the chi-square test or Fisher’s exact test, as appropriate. For comparisons across more than two independent groups, the Kruskal–Wallis test was used when the variable of interest was ordinal or not normally distributed. This approach was used, for example, to compare O-SIMAS scores across operated spinal segments. Associations between ordinal or non-normally distributed variables were explored using Spearman’s rank correlation coefficient, which assesses monotonic relationships without assuming linearity or normal distribution. Effect sizes for binary outcomes were reported as odds ratios with 95% confidence intervals.

Interrater agreement for O-SIMAS was assessed using the independent pre-consensus ratings. For the full ordinal 0–5 scale, pairwise quadratic-weighted Cohen’s kappa coefficients were computed for each rater pair. Exact agreement and agreement within one grade were also calculated. For the dichotomized classification of low- versus high-grade artifacts, multirater agreement was assessed using Fleiss’ kappa.

All primary analyses of artifact burden used the final consensus O-SIMAS grade. Exploratory logistic regression analyses were performed for selected binary endpoints. Binary outcomes were coded as present versus absent. Univariable logistic regression was first used to estimate crude associations between individual predictors and the outcome. These models included the association between O-SIMAS grade and local recurrence status, as well as predictors of high-grade artifact status, defined as O-SIMAS grades 3–5 versus grades 0–2. Odds ratios with 95% confidence intervals were reported for each model. An exploratory multivariable logistic regression model was then constructed for high-grade artifact status. The dependent variable was high-grade artifact status, defined as O-SIMAS grades 3–5 versus grades 0–2. The independent variables were implant material, vertebral body prosthesis or reconstruction, operated spinal segment, and Gertzbein–Robbins grade [29]. Because the cervical subgroup was small and all cervical cases were classified as high-grade, cervical cases were excluded from the multivariable model to avoid instability due to complete separation.

Overall survival and progression-free survival were estimated using the Kaplan–Meier method. For overall survival, death from any cause was treated as the event, and patients alive at the last available follow-up were censored at the date of last contact. For progression-free survival, local recurrence or death, whichever occurred first, was treated as the event, and patients without either event were censored at the date of last documented clinical or radiographic follow-up. Kaplan–Meier curves were generated separately for the CFR-PEEK and titanium groups, and median survival times were reported when estimable. Between-group differences in survival distributions were assessed using the log-rank test. Local recurrence was additionally analyzed using cumulative incidence functions, with death without prior local recurrence treated as a competing event; group differences were assessed using Gray’s test. Missing data were not imputed, and complete-case analysis was performed for each endpoint. All tests were two-sided, and *p* < 0.05 was considered statistically significant.

### 2.8. Ethics

Given the retrospective design of the present analysis and the use of anonymized data, formal ethics committee approval was not required under local institutional policy. The study was conducted in accordance with applicable institutional standards for retrospective analyses of anonymized clinical data.

## 3. Results

### 3.1. Patient Cohort and Baseline Characteristics

The primary comparative cohort consisted of 78 patients treated with instrumented stabilization, including 33 who received CFR-PEEK instrumentation and 45 who received titanium instrumentation. The single case treated with mixed instrumentation was excluded from the main analysis. Median age for the overall cohort was 67.5 years (interquartile range [IQR] 59.0–73.0), including 68.0 years (IQR 60.0–73.0) in the CFR-PEEK group and 66.0 years (IQR 57.0–72.0) in the titanium group. Overall, 46 patients (59.0%) were female, and 32 (41.0%) were male. Most patients had a relatively preserved performance status, with Eastern Cooperative Oncology Group (ECOG) performance status 0–II in 61 patients (78.2%) and ECOG III–IV in 17 patients (21.8%). The most common histopathological diagnoses were kidney carcinoma (19 patients), lung cancer (16 patients), and breast cancer (13 patients). The other category comprised 11 patients. Smaller groups included sarcoma (4 patients), colorectal cancer (3 patients), prostate cancer (3 patients), germ cell tumors (2 patients), melanoma (2 patients), and hepatocellular carcinoma (2 patients), whereas esophageal, pancreatic, and salivary gland cancers were represented by one patient each. Regarding operative strategy, separation-based procedures were the most common, performed in 35 patients (44.9%), followed by laminectomy in 20 patients (25.6%), corpectomy-based procedures in 19 patients (24.4%), and stabilization-only in 4 patients (5.1%). The median number of stabilized levels was 5.0 (IQR 4.0–5.0) in the overall cohort, 5.0 (IQR 5.0–5.0) in the CFR-PEEK group, and 5.0 (IQR 3.0–5.0) in the titanium group. Postoperative radiotherapy was administered in most patients. Overall, 20 patients (25.6%) received no radiotherapy, 23 (29.5%) received non-SBRT radiotherapy, and 35 (44.9%) received SBRT. Vertebral body prosthesis or reconstruction at the level of the operated vertebra was present in 15 patients (19.2%). Prior systemic therapy was documented in 27 patients (34.6%), whereas 50 (64.1%) had not received systemic treatment. The most common systemic modality was chemotherapy (22 patients, 28.2%), followed by hormone therapy (8 patients, 10.3%) and immunotherapy (3 patients, 3.8%). Bone cement augmentation was used in 17 of 79 patients (21.5%), including one mixed-instrumentation case, which was removed from the other analysis. Among patients who received cement augmentation, instrumentation was carbon-based in 11 cases (64.7%), titanium-based in 5 cases (29.4%), and mixed titanium–carbon in 1 case (5.9%). Thus, when the mixed construct was counted by material presence, carbon instrumentation was present in 12 of 17 cases (70.6%), whereas titanium was present in 6 of 17 cases (35.3%).

O-SIMAS scale scores were available in 9 of 17 cement-augmented patients (52.9%), while 8 patients (47.1%) had no MRI follow-up. Among evaluable patients, the O-SIMAS score distribution was as follows: grade 0 in 3 patients (33.3%), grade 1 in 2 patients (22.2%), grade 2 in 2 patients (22.2%), grade 3 in 1 patient (11.1%), grade 4 in 0 patients (0%), and grade 5 in 1 patient (11.1%).

Full baseline characteristics are summarized in Table 2.

### 3.2. Postoperative MRI Assessability and Artifact Burden

**Figure 2:**
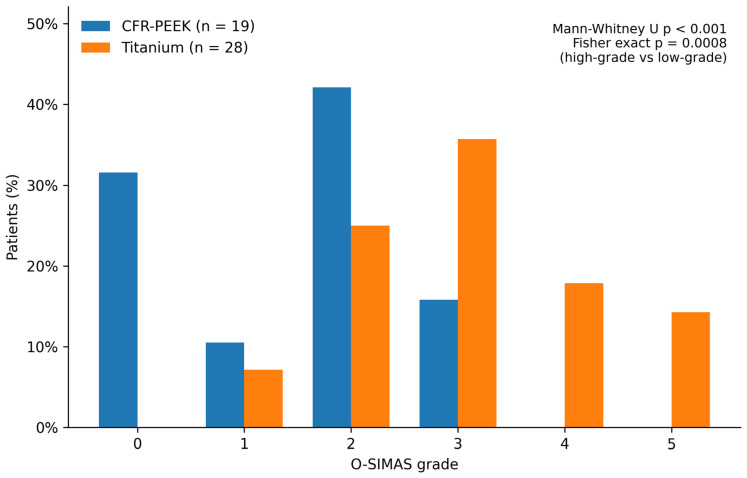
Distribution of O-SIMAS grades according to implant material. The CFR-PEEK group showed a clear shift toward lower artifact grades, whereas the titanium group showed higher grades predominating. The difference in score distribution was statistically significant (Mann–Whitney U test, *p* < 0.001). High-grade artifacts (O-SIMAS grades 3–5) were also significantly more frequent in the titanium group than in the CFR-PEEK group (Fisher’s exact test, *p* = 0.0008).

Postoperative MRI examinations were available for review in 47 patients, including 19 treated with CFR-PEEK instrumentation and 28 treated with titanium instrumentation. For each MRI-evaluable patient, a final consensus O-SIMAS grade was assigned after review of the independent ratings. This consensus grade represented the primary analytical artifact score and was used for all artifact-related group comparisons between CFR-PEEK and titanium instrumentation, dichotomized low-grade versus high-grade artifact analyses, and logistic regression models evaluating predictors of high-grade artifact status. Independent pre-consensus ratings from the three raters were used only for interrater agreement analyses. In the overall MRI-evaluable cohort, the median O-SIMAS score was 2.0 (IQR 2.0–3.0; range 0–5). Because O-SIMAS is an ordinal 0–5 grading system and its distribution was not assumed to be symmetrical, artifact burden was summarized primarily using medians, interquartile ranges, ranges, and grade distributions rather than mean ± standard deviation. Artifact burden was significantly lower in the CFR-PEEK group than in the titanium group, with median scores of 2.0 (IQR 0.0–2.0; range 0–3) versus 3.0 (IQR 2.0–4.0; range 1–5), respectively (Mann–Whitney U = 88.0, *p* < 0.001). The score distribution was shifted toward lower grades in the CFR-PEEK group: no CFR-PEEK cases were assigned a score of 4 or 5, whereas no titanium cases received a score of 0 (Figure 2).

When dichotomized, low-grade artifacts (O-SIMAS grades 0–2) were observed in 25 of 47 cases (53.2%), and high-grade artifacts (grades 3–5) in 22 of 47 cases (46.8%). This distribution differed markedly by implant material. In the CFR-PEEK group, 16 of 19 patients (84.2%) had low-grade artifacts and 3 of 19 (15.8%) had high-grade artifacts, compared with 9 of 28 (32.1%) and 19 of 28 (67.9%), respectively, in the titanium group (Fisher’s exact test, *p* = 0.0008).

Both the Gertzbein–Robbins classification [29] and the O-SIMAS score were available for 46 cases. Grade A was found in 41 cases (89.1%), and grade B in 5 cases (10.9%), with no occurrences of grades C, D, or E. The O-SIMAS scores did not show a significant difference between Gertzbein–Robbins grades A and B (median 2.0 [IQR 2.0–3.0] in both groups; Mann–Whitney U = 92.0, *p* = 0.717). Additionally, there was no significant monotonic correlation between the two scales (Spearman’s rho = 0.057, *p* = 0.708).

Artifact burden also appeared to vary by spinal segment. In the full MRI-evaluable cohort, including cervical cases, median O-SIMAS scores were 4.0 (IQR 4.0–4.0) for the cervical segment (*n* = 3), 3.0 (IQR 2.0–3.0) for the thoracic segment (*n* = 22), 2.0 (IQR 1.0–2.75) for the thoracolumbar segment (*n* = 14), and 2.0 (IQR 1.0–2.25) for the lumbar segment (*n* = 8). The overall between-segment difference was borderline significant (Kruskal–Wallis *p* = 0.051). Because the cervical subgroup was very small and potentially disproportionately influential, a sensitivity analysis was performed after excluding cervical cases; in this restricted analysis, no significant differences were observed across thoracic, thoracolumbar, and lumbar procedures (Kruskal–Wallis *p* = 0.207).

The use of vertebral body prosthesis was associated with a higher MRI artifact burden. Patients treated with vertebral body prosthesis had a median O-SIMAS score of 4.0 (IQR 2.75–4.25), compared with 2.0 (IQR 2.0–3.0) in patients without prosthesis (Mann–Whitney U = 80.5, *p* = 0.029). High-grade artifacts were observed in 6 of 8 patients (75.0%) with a prosthesis and in 16 of 39 (41.0%) without a prosthesis, although this difference was not significant in the binary analysis (Fisher’s exact test, *p* = 0.123).

No association was found between O-SIMAS grade and local recurrence. Among MRI-evaluable patients, the median O-SIMAS score was 2.5 (IQR 2.0–3.0) in patients with local recurrence and 2.0 (IQR 2.0–3.0) in those without recurrence (Mann–Whitney U = 153.5, *p* = 0.953). Likewise, no significant monotonic correlation was observed between O-SIMAS grade and local recurrence status (Spearman’s rho = 0.011, *p* = 0.943). In a univariate logistic model, a 1-point increase in O-SIMAS was not associated with local recurrence (OR 0.98, 95% CI 0.57–1.70; *p* = 0.947).

An exploratory multivariable logistic regression model was then constructed using high-grade artifact status (O-SIMAS grades 3–5) as the dependent variable and implant material, vertebral body prosthesis, operated spinal segment, and Gertzbein–Robbins grade as predictors. Because the cervical subgroup was very small and all cervical cases were classified as high-grade, cervical cases were excluded from the multivariable model to avoid instability due to complete separation; thus, the model was limited to noncervical cases with available Gertzbein–Robbins data. In this analysis, titanium instrumentation remained independently associated with high-grade artifacts (OR 16.09, 95% CI 2.47–104.97; *p* = 0.004). Compared with lumbar cases, thoracic procedures were also associated with a higher likelihood of high-grade artifacts (OR 28.24, 95% CI 2.21–360.90; *p* = 0.010), whereas thoracolumbar location was not significantly associated with the outcome (OR 3.19, 95% CI 0.31–32.73; *p* = 0.330). Vertebral body prosthesis showed a strong but non-significant trend toward higher odds of high-grade artifact (OR 9.36, 95% CI 0.68–128.42; *p* = 0.094), while Gertzbein–Robbins grade B was not significantly associated with artifact severity (OR 0.12, 95% CI 0.01–2.09; *p* = 0.146) (Table 3). Given the limited sample size and wide confidence intervals, these multivariable findings should be interpreted as exploratory.

Complete O-SIMAS ratings from all 3 independent raters were available in 46 cases. Pairwise inter-rater agreement on the full ordinal 0–5 scale was substantial, with quadratic-weighted Cohen’s kappa values of 0.728 (95% CI 0.531–0.856), 0.769 (95% CI 0.591–0.878), and 0.808 (95% CI 0.647–0.910) for the 3 rater pairs, respectively. Exact agreement among all 3 raters was observed in 17 of 46 cases (37.0%), whereas agreement within 1 grade was achieved in 36 of 46 cases (78.3%). After dichotomization into low-grade (grades 0–2) and high-grade (grades 3–5) artifacts, multirater agreement was moderate (Fleiss’ kappa 0.486, 95% CI 0.295–0.667), with complete agreement in 29 of 46 cases (63.0%).

### 3.3. Early Postoperative Outcomes

Early postoperative outcomes were comparable between the CFR-PEEK and titanium groups, with no statistically significant intergroup differences. These findings indicate that the lower MRI artifact burden observed with CFR-PEEK instrumentation was not associated with an increased rate of early postoperative morbidity in this cohort. New postoperative neurological deficits occurred in 12 of 78 patients (15.4%), including 3 of 33 patients (9.1%) in the CFR-PEEK group and 9 of 45 patients (20.0%) in the titanium group (*p* = 0.221). Reoperation was required in 9 of 78 patients (11.5%), including 3 of 33 patients (9.1%) treated with CFR-PEEK and 6 of 45 patients (13.3%) treated with titanium instrumentation (*p* = 0.726). Wound complications were observed in 7 of 78 patients (9.0%), including 4 of 33 patients (12.1%) in the CFR-PEEK group and 3 of 45 patients (6.7%) in the titanium group (*p* = 0.448). The median length of hospital stay was 8.0 days (IQR 5.0–12.5) overall, 8.0 days (IQR 6.0–13.0) in the CFR-PEEK group, and 7.5 days (IQR 5.0–12.0) in the titanium group (*p* = 0.362); length-of-stay data were available for 75 patients.

Among the 12 patients with new postoperative neurological deficits, motor deterioration predominated. Isolated sensory deficits were defined as abnormalities of superficial or deep sensation, including radicular paresthesias. Five patients developed postoperative weakness, uniformly limited to a 1-grade decline on the Medical Research Council (MRC) scale for muscle strength; in all of these cases, muscle strength recovered to the preoperative level during follow-up. In contrast, the three patients categorized as having paralysis represented a distinct clinical subgroup characterized by rapidly progressive neurological deterioration within hours before surgery and severe preoperative motor impairment, with muscle strength graded at 3/5 or lower on the MRC scale. None of these patients regained their pre-deterioration neurological status (Table 4).

### 3.4. Oncologic Outcomes

**Figure 3:**
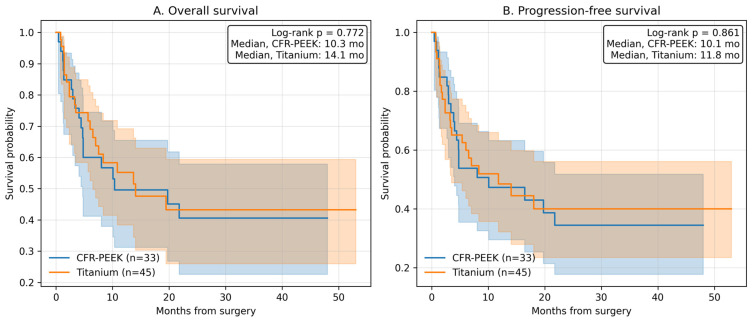
Kaplan–Meier estimates of overall survival (**A**) and progression-free survival (**B**) according to implant material. Progression-free survival was defined as the time from surgery to local recurrence or death, whichever occurred first. *p* values were calculated using the log-rank test. Abbreviations: CFR-PEEK, carbon-fiber-reinforced polyetheretherketone.

During follow-up, 11 of 78 patients (14.1%) developed local recurrence, including 3 of 33 patients (9.1%) in the CFR-PEEK group and 8 of 45 patients (17.8%) in the titanium group. Kaplan–Meier analysis showed a median overall survival (OS) of 14.1 months in the overall cohort. Median OS was 10.3 months in the CFR-PEEK group and 14.1 months in the titanium group, with no significant between-group difference (log-rank *p* = 0.772). Median progression-free survival (PFS), defined as the interval from surgery to local recurrence or death, whichever occurred first, was 10.1 months in the overall cohort, 10.1 months in the CFR-PEEK group, and 11.8 months in the titanium group, with no significant between-group difference (log-rank *p* = 0.861) (Figure 3). Thus, although CFR-PEEK instrumentation was associated with improved postoperative MRI assessability, no statistically significant difference in oncological follow-up outcomes was observed between implant groups.

**Figure 4:**
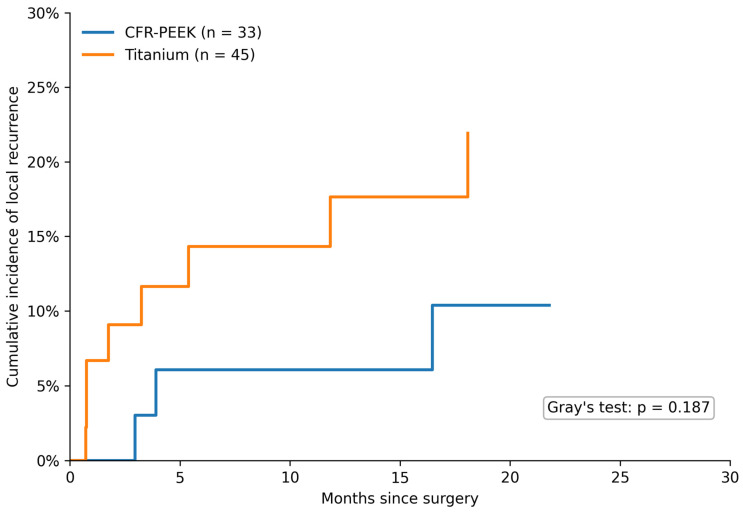
Cumulative incidence of local recurrence according to implant material, with death without prior local recurrence treated as a competing event. The cumulative incidence of local recurrence was numerically lower in the CFR-PEEK group than in the titanium group, but the difference was not statistically significant (Gray’s test, *p* = 0.187). Abbreviations: CFR-PEEK, carbon-fiber-reinforced polyetheretherketone.

Local recurrence was additionally analyzed in a competing-risks framework, with death without prior local recurrence treated as a competing event. In this analysis, the cumulative incidence of local recurrence remained numerically lower in the CFR-PEEK group than in the titanium group, but the difference did not reach statistical significance (Gray’s test, *p* = 0.187). The estimated cumulative incidence of local recurrence at 12 months was 6.1% in the CFR-PEEK group and 17.7% in the titanium group, increasing to 10.4% and 22.1%, respectively, at 24 months (Figure 4). Overall, these findings indicate that although local recurrence occurred numerically less often after CFR-PEEK instrumentation, no statistically significant between-group differences in oncologic time-to-event outcomes were observed during the available follow-up period (Table 5). However, because the number of local recurrence events was limited, these oncological time-to-event analyses were considered exploratory.

## 4. Discussion

In this single-center cohort comparison, CFR-PEEK instrumentation was associated with a substantially lower burden of postoperative MRI artifacts than titanium, whereas early postoperative and oncological outcomes did not differ significantly between groups. The artifact-related difference was evident both on the full ordinal O-SIMAS scale and after dichotomization into low- and high-grade artifacts. The study-specific O-SIMAS grading system also demonstrated substantial interrater agreement, supporting its use as a structured method for assessing the extent and diagnostic relevance of postoperative implant-related MRI artifacts. These findings are consistent with previous reports suggesting that CFR-PEEK improves postoperative imaging interpretability without compromising short-term clinical outcomes [19,20,21,22,23,24,25,26,27,28].

The present findings extend previous work on CFR-PEEK instrumentation in spinal oncology. Earlier clinical series and comparative studies primarily demonstrated the feasibility, safety, and potential radiotherapy-planning advantages of CFR-PEEK constructs [19,23,24,25]. More recent studies have provided comparative or quantitative evidence that CFR-PEEK may reduce imaging artifacts and facilitate postoperative radiotherapy workflows when compared with titanium instrumentation [20,21,26]. Systematic reviews have also emphasized the potential imaging and radiotherapy benefits of CFR-PEEK, while noting that available evidence remains limited by retrospective designs, heterogeneous endpoints, and relatively small cohorts [22,27]. In line with these reports, our study found no significant differences in early postoperative outcomes between CFR-PEEK and titanium instrumentation, but demonstrated a significant reduction in postoperative MRI artifact burden with CFR-PEEK. The main additional contribution of the present study is the use of a structured, anatomy-based grading system to distinguish artifacts that remain adjacent to the instrumentation from those that compromise clinically relevant regions at the level of operation.

The main implication of this study is not only that CFR-PEEK produces fewer artifacts than titanium, but that it reduces diagnostically relevant artifacts at the operated level. This is clinically important because postoperative MRI in spinal oncology is used to evaluate the epidural space, spinal canal, operated vertebral body, and reconstruction site during surveillance [4,5,8,9,28,30]. Titanium implants may impair follow-up imaging and radiotherapy planning because of susceptibility artifacts, whereas CFR-PEEK was developed to mitigate these limitations [13,14,15,16,17,18,25,26]. From an imaging perspective, the lower artifact burden associated with CFR-PEEK may improve visualization of the epidural space, spinal canal, operated vertebral body, and reconstruction site during postoperative surveillance. From a radiotherapy perspective, improved image quality may facilitate target-volume and organ-at-risk delineation, reduce uncertainty during postoperative treatment planning, and support more reliable integration of MRI findings into stereotactic radiotherapy workflows. By grading artifacts according to anatomical extent and diagnostic relevance, O-SIMAS may capture this problem more precisely than a simple binary assessment of MRI assessability [13,15,26].

Most prior studies have described the imaging advantages of CFR-PEEK qualitatively or in broad binary terms, without a formalized anatomical framework for determining whether artifacts affect structures relevant to residual or recurrent disease [19,20,21,22,23,24,25,26,27,28]. O-SIMAS should therefore be viewed as an initial, study-specific attempt to standardize postoperative MRI artifact assessment in spinal oncology. Interrater agreement for the full ordinal scale was substantial, although agreement after dichotomization was only moderate, likely reflecting the importance of the grade 2/3 threshold. This transition is clinically relevant because it distinguishes artifacts adjacent to the instrumentation from artifacts extending to the operated level. Future validation studies should specifically assess the reproducibility and clinical meaning of this boundary.

Artifact burden appeared to vary by spinal segment in the full MRI-evaluable cohort, but this pattern was mainly influenced by the small cervical subgroup. After excluding cervical cases, no significant between-segment differences remained. This finding should therefore be interpreted cautiously, and larger multicenter cohorts are needed to determine whether cervical instrumentation presents specific challenges for MRI follow-up.

The radiological advantage of CFR-PEEK was not associated with significant differences in early postoperative outcomes. Rates of new neurological deficits, reoperations, wound complications, and length of hospital stay were comparable between groups, consistent with previous comparative reports suggesting that CFR-PEEK provides imaging and radiotherapy-planning advantages while maintaining acceptable perioperative safety [19,20,21,22,23,24,25,26,27].

Although CFR-PEEK instrumentation was associated with a clear radiological advantage, this did not translate into statistically significant differences in oncological outcomes during the available follow-up period. Local recurrence occurred numerically less often after CFR-PEEK instrumentation, and competing-risks analysis showed the same directional trend; however, these differences were not statistically significant. The study was not powered to determine whether implant material influences local control, OS, or PFS. Therefore, the observed recurrence difference should be considered hypothesis-generating rather than evidence of superior oncological efficacy. Implant-related artifact is most directly relevant to surveillance, recurrence detectability, target delineation, and radiotherapy planning, rather than to tumor biology itself. This interpretation is consistent with recent comparative data suggesting that implant material may influence recurrence detection rather than directly improving tumor control [28].

This study has several limitations. Its retrospective, single-center design carries risks of selection bias, information bias, and unmeasured confounding. Implant allocation was not randomized and may have been influenced by anatomical, oncological, technical, economic, or logistical factors not fully captured in the dataset. Postoperative MRI was not available for all patients, which may have introduced selection bias into the MRI-evaluable subgroup.

The number of oncological events was limited, and the study was underpowered to detect meaningful differences in local recurrence, PFS, or OS. O-SIMAS is an original, study-specific scale and has not undergone external validation. Although interrater agreement was substantial for the full ordinal scale, future studies should confirm its reproducibility, diagnostic thresholds, and clinical relevance, particularly the distinction between grade 2 and grade 3 artifacts. Finally, the multivariable artifact model was exploratory and limited by the small MRI-evaluable cohort, subgroup exclusions, and wide confidence intervals.

Despite these limitations, the study addresses a clinically relevant problem at the intersection of surgery, postoperative radiotherapy, and MRI surveillance. By combining comparative clinical data, structured artifact grading, interrater agreement analysis, and competing-risk analysis for local recurrence, this study provides an integrated assessment of implant-related imaging effects in spinal oncology. Overall, CFR-PEEK instrumentation appears to offer a clear postoperative MRI advantage over titanium by reducing high-grade artifacts that impair assessment at the operated level. O-SIMAS provides a practical initial framework for standardizing this assessment and warrants external validation.

## 5. Conclusions

In this retrospective single-center cohort study, carbon fiber-reinforced PEEK instrumentation was associated with significantly lower postoperative MRI artifact burden and improved MRI assessability compared with titanium instrumentation in patients treated surgically for metastatic spinal disease. This advantage was demonstrated using the study-specific O-SIMAS grading system and was particularly evident in the lower frequency of high-grade artifacts compromising clinically relevant regions at the operated level. Early postoperative outcomes were comparable between groups, with no statistically significant differences in neurological deficits, reoperations, wound complications, or length of hospital stay. Local recurrence, progression-free survival, and overall survival also did not differ significantly between implant groups; therefore, no oncological benefit of CFR-PEEK can be inferred from the present data. O-SIMAS may provide a practical framework for structured postoperative MRI artifact assessment in spinal oncology, but it requires external validation in larger prospective cohorts.

## Figures and Tables

**Table 1:** Summary of selected previous studies on carbon fiber-reinforced PEEK instrumentation in spinal oncology. The table highlights the design, population, focus, and relevance of existing clinical, imaging, and review studies to the present analysis of postoperative MRI artifact burden.

Study	Design/Population	Main Focus	Key Relevance to Present Study
Boriani et al. [25]	Preliminary clinical report on carbon-fiber fixation in spine tumors	Feasibility and early clinical experience	Early evidence supporting the use of CFR-PEEK constructs in spinal oncology
Cofano et al. [19]	Comparative clinical study of CFR-PEEK versus titanium implants in spinal metastases	Safety and effectiveness of a carbon-based strategy	Supports clinical feasibility of CFR-PEEK compared with titanium instrumentation
Neal et al. [24]	Retrospective series in spinal oncology	Technique, feasibility, and clinical outcomes	Demonstrates practical use of CFR-PEEK instrumentation in oncologic spine surgery
Joerger et al. [23]	Single-center cohort of spinal neoplasms	Safety and efficacy of CFR-PEEK pedicle screw instrumentation	Provides large-cohort safety data for CFR-PEEK in spinal tumor surgery
Khan et al. [22]	Systematic review of primary and metastatic spine tumors	Implant complications and radiotherapy-related benefits	Highlights potential imaging/radiotherapy advantages and the need for stronger comparative data
Kumar et al. [20]	Comparative study of CFR-PEEK versus titanium implants	Clinical outcomes and postoperative radiotherapy planning	Directly compares implant materials and supports the relevance of postoperative planning advantages
de Almeida et al. [21]	Quantitative imaging study	MRI artifacts in CFR-PEEK thoracolumbar constructs before spine stereotactic radiosurgery	Supports the importance of objective artifact assessment for postoperative SBRT workflows
Ward et al. [28]	Case-matched comparative series	Instrumentation material and local recurrence	Suggests that implant material may influence recurrence detection and oncological follow-up
Zhang et al. [27]	Systematic review and meta-analysis	CFR-PEEK versus titanium implants for spinal tumors	Provides recent pooled evidence on outcomes, complications, and radiotherapy-related effects

**Table 2:** Baseline characteristics and treatment variables in the primary comparative cohort treated with CFR-PEEK or titanium instrumentation. Values are presented as *n* (%) or median (IQR). Abbreviations: CFR-PEEK = carbon-fiber-reinforced polyetheretherketone; ECOG = Eastern Cooperative Oncology Group; IQR = interquartile range; SBRT = stereotactic body radiotherapy. * Systemic treatment modalities were counted nonexclusively. *p* values are provided descriptively to summarize baseline comparability between implant groups and should not be interpreted as evidence of unbiased allocation or causal equivalence.

Characteristic	Overall (*n* = 78)	CFR-PEEK (*n* = 33)	Titanium (*n* = 45)	*p* Value
Age, median (IQR)	67.5 (59.0–73.0)	68.0 (60.0–73.0)	66.0 (57.0–72.0)	0.504
Sex				
Female	46 (59.0%)	21 (63.6%)	25 (55.6%)	0.495
Male	32 (41.0%)	12 (36.4%)	20 (44.4%)	
ECOG				
0–II	61 (78.2%)	27 (81.8%)	34 (75.6%)	0.587
III–IV	17 (21.8%)	6 (18.2%)	11 (24.4%)	
Histopathology				
Kidney	19 (24.4%)	9 (27.3%)	10 (22.2%)	0.184
Lung cancer	16 (20.5%)	6 (18.8%)	10 (26.7%)	
Breast cancer	13 (16.7%)	5 (15.2%)	8 (17.8%)	
Other	11 (14.1%)	4 (12.1%)	7 (15.6%)	
Sarcoma	4 (5.1%)	4 (12.1%)	0 (0.0%)	
Colorectal cancer	3 (3.8%)	1 (3.0%)	2 (4.4%)	
Prostate cancer	3 (3.8%)	1 (3.0%)	2 (4.4%)	
Germ cell tumor	2 (2.6%)	2 (6.1%)	0 (0.0%)	
Melanoma	2 (2.6%)	0 (0.0%)	2 (4.4%)	
Hepatocellular carcinoma	2 (2.6%)	1 (3.0%)	1 (2.2%)	
Esophageal cancer	1 (1.3%)	1 (3.0%)	0 (0.0%)	
Pancreatic cancer	1 (1.3%)	0 (0.0%)	1 (2.2%)	
Salivary gland cancer	1 (1.3%)	1 (3.0%)	0 (0.0%)	
Implant material				
CFR-PEEK	33 (42.3%)			
Titanium	45 (57.7%)			
Stabilization technique				
Percutaneous pedicle screw fixation	48 (61.5%)	19 (57.6%)	29 (64.4%)	0.106
Open pedicle screw fixation	26 (33.3%)	14 (42.4%)	12 (26.7%)	
Anterior cervical corpectomy	4 (5.1%)	0 (0.0%)	4 (8.9%)	
Operation type				
Separation surgery	35 (44.9%)	15 (45.5%)	20 (44.4%)	0.864
Corpectomy surgery	19 (24.4%)	9 (27.3%)	10 (22.2%)	
Laminectomy, posterior decompression	20 (25.6%)	7 (21.2%)	13 (28.9%)	
Stabilization only	4 (5.1%)	2 (6.1%)	2 (4.4%)	
Stabilized levels, median (IQR)	5.0 (4.0–5.0)	5.0 (5.0–5.0)	5.0 (3.0–5.0)	0.817
Postoperative radiotherapy				
No RT	20 (25.6%)	12 (36.4%)	8 (17.8%)	0.118
Non-SBRT RT	23 (29.5%)	10 (30.3%)	13 (28.9%)	
SBRT	35 (44.9%)	11 (33.3%)	24 (53.3%)	
Vertebral body reconstruction				
Yes	15 (19.2%)	6 (18.2%)	9 (20.0%)	1.000
No	63 (80.8%)	27 (81.8%)	36 (80.0%)	
Prior systemic therapy				
Yes	27 (34.6%)	13 (39.4%)	14 (31.1%)	0.346
No	50 (64.1%)	19 (57.6%)	31 (68.9%)	
Missing	1 (1.3%)	1 (3.0%)	0 (0.0%)	
Systemic treatment modalities *				
Chemotherapy	22 (28.2%)	11 (33.3%)	11 (24.4%)	0.450
Hormone therapy	8 (10.3%)	3 (9.1%)	5 (11.1%)	
Immunotherapy	3 (3.8%)	2 (6.1%)	1 (2.2%)	

**Table 3:** Univariable and multivariable predictors of high-grade postoperative MRI artifacts. Odds ratios are shown for high-grade artifacts, defined as O-SIMAS grades 3–5. The multivariable model included implant material, vertebral body prosthesis, operated spinal segment, and Gertzbein–Robbins grade and was restricted to noncervical cases. CFR-PEEK was used as the reference category for implant material, lumbar location, and Gertzbein–Robbins grade A for screw placement accuracy. Values are presented as OR (95% CI). Abbreviations: CFR-PEEK = carbon fiber-reinforced polyetheretherketone; CI = confidence interval; O-SIMAS = Oncologic Spinal Instrumentation MRI Artifact Score; OR = odds ratio.

Variable	Comparison	Univariable OR (95% CI)	*p* Value	Multivariable OR (95% CI)	*p* Value
Implant material	Titanium vs. CFR-PEEK	8.33 (1.88–36.97)	0.005	16.09 (2.43–104.12)	0.004
Vertebral body prosthesis	Yes vs. no	2.20 (0.33–14.79)	0.417	9.36 (0.65–127.58)	0.102
Operated segment	Thoracolumbar vs. lumbar	1.20 (0.17–8.66)	0.857	3.17 (0.31–32.66)	0.331
Operated segment	Thoracic vs. lumbar	4.50 (0.72–28.15)	0.108	28.24 (2.17–354.00)	0.011
Gertzbein–Robbins grade	B vs. A	0.88 (0.13–5.87)	0.891	0.12 (0.01–2.13)	0.149

**Table 4:** Early postoperative outcomes in the comparative cohort, including the distribution of new postoperative neurological deficits. CFR-PEEK, carbon fiber-reinforced polyetheretherketone; IQR, interquartile range.

Variable	Overall	CFR-PEEK (*n* = 33)	Titanium (*n* = 45)	*p* Value
New postoperative neurological deficits, *n* (%)	12/78 (15.4)	3/33 (9.1)	9/45 (20.0)	0.221
Mild postoperative motor worsening	5	2	3	—
Persistent severe motor deficit in patients with rapidly progressive preoperative neurological deterioration	3	1	2	—
Isolated sensory disturbances	4	0	4	—
Reoperation, *n* (%)	9/78 (11.5)	3/33 (9.1)	6/45 (13.3)	0.726
Wound complications, *n* (%)	7/78 (9.0)	4/33 (12.1)	3/45 (6.7)	0.448
Length of hospital stay, days, median (IQR)	8.0 (5.0–12.5)	8.0 (6.0–13.0)	7.5 (5.0–12.0)	0.362

**Table 5:** Oncologic follow-up and time-to-event outcomes in the comparative cohort, including cumulative incidence estimates for local recurrence with death treated as a competing event. CFR-PEEK, carbon-fiber-reinforced polyetheretherketone; OS, overall survival; PFS, progression-free survival.

Variable	Overall (*n* = 78)	CFR-PEEK (*n* = 33)	Titanium (*n* = 45)	*p* Value
Local recurrence, *n* (%)	11 (14.1%)	3 (9.1%)	8 (17.8%)	0.335
Median overall survival (OS), months	14.1	10.3	14.1	0.772
Median progression-free survival (PFS), months	10.1	10.1	11.8	0.861
12-month cumulative incidence of local recurrence, %	12.6	6.1	17.7	0.187
24-month cumulative incidence of local recurrence, %	16.9	10.4	22.1	0.187

## Data Availability

The data supporting the findings of this study are not publicly available due to privacy and ethical restrictions related to patient-level clinical information. Anonymized data may be made available by the corresponding author upon reasonable request.

## Supplementary Materials

The following supporting information can be downloaded at: https://www.mdpi.com/article/10.3390/cancers18142199/s1, Table S1: STROBE Statement—Checklist of items that should be included in reports of cohort studies.

## Abbreviations

CFR-PEEK	carbon fiber-reinforced polyetheretherketone
CI	confidence interval
ECOG	Eastern Cooperative Oncology Group
IQR	interquartile range
MRI	magnetic resonance imaging
MRC	Medical Research Council
O-SIMAS	Oncologic Spinal Instrumentation MRI Artifact Score
OR	odds ratio
OS	overall survival
PFS	progression-free survival
PMMA	polymethylmethacrylate
RANO	Response Assessment in Neuro-Oncology
SBRT	stereotactic body radiotherapy
SPINO	Spine Response Assessment in Neuro-Oncology
